# British randomised controlled trial of AV and VV optimization (“BRAVO”) study: rationale, design, and endpoints

**DOI:** 10.1186/1471-2261-14-42

**Published:** 2014-04-03

**Authors:** Zachary I Whinnett, S M Afzal Sohaib, Siana Jones, Andreas Kyriacou, Katherine March, Emma Coady, Jamil Mayet, Alun D Hughes, Michael Frenneaux, Darrel P Francis

**Affiliations:** 1 International Centre for Circulatory Health, National Heart and Lung Institute, Imperial College London, 59-61 North Wharf Road, London W2 1LA, UK; 2 Institute of Cardiovascular Sciences, University College London, Gower Street, London WC1E 6BT, UK; 3 Institute of Medical Sciences, University of Aberdeen, Foresterhill, Aberdeen AB25 2ZD, UK

**Keywords:** Heart failure, Cardiac resynchronization therapy, Optimization

## Abstract

**Background:**

Echocardiographic optimization of pacemaker settings is the current standard of care for patients treated with cardiac resynchronization therapy. However, the process requires considerable time of expert staff. The BRAVO study is a non-inferiority trial comparing echocardiographic optimization of atrioventricular (AV) and interventricular (VV) delay with an alternative method using non-invasive blood pressure monitoring that can be automated to consume less staff resources.

**Methods/Design:**

BRAVO is a multi-centre, randomized, cross-over, non-inferiority trial of 400 patients with a previously implanted cardiac resynchronization device. Patients are randomly allocated to six months in each arm. In the echocardiographic arm, AV delay is optimized using the iterative method and VV delay by maximizing LVOT VTI. In the haemodynamic arm AV and VV delay are optimized using non-invasive blood pressure measured using finger photoplethysmography. At the end of each six month arm, patients undergo the primary outcome measure of objective exercise capacity, quantified as peak oxygen uptake (VO_2_) on a cardiopulmonary exercise test. Secondary outcome measures are echocardiographic measurement of left ventricular remodelling, quality of life score and N-terminal pro B-type Natriuretic Peptide (NT-pro BNP). The study is scheduled to complete recruitment in December 2013 and to complete follow up in December 2014.

**Discussion:**

If exercise capacity is non-inferior with haemodynamic optimization compared with echocardiographic optimization, it would be proof of concept that haemodynamic optimization is an acceptable alternative which has the potential to be more easily implemented.

**Trial registration:**

Clinicaltrials.gov NCT01258829

## Background

Onset of biventricular pacing results in immediate improvements in cardiac function which is manifest as an improvement in haemodynamic parameters [1-3] including increased peak rate of rise of intraventricular pressure, [4] an increase in stroke volume [5], and higher systemic arterial blood pressure [4,6]. Longer-term effects include improvements in clinical outcome measures including exercise capacity, reductions in LV volumes, quality of life measures [7-9] and NT-pro BNP [10]. Over even longer time frames, biventricular pacing has been shown to reduce hospitalizations and mortality [11,12].

Although the precise mechanism of clinical benefit from biventricular pacing is not yet adequately established, it is clear that it can only affect cardiac function by changing the timing of the electrical signals initiating cardiac contraction. Just as switching on and off CRT creates immediate haemodynamic effects, so does changing the AV and VV timings [1,2,13]. Since the benefits must come from the change in timings, it might matter what timings are programmed.

The precise AV and VV delays that maximise haemodynamic measurements vary between patients, perhaps because of the complexity of the disease which has many elements present in varying degrees in different individuals, and also perhaps because of anatomical variations in lead position [1,14-16].

Tailoring biventricular pacing therapy to an individual by optimizing the AV and VV delay settings of the device has long been considered a mechanistically plausible way to maximise its clinical benefit. AV delay optimization was performed in the landmark studies of biventricular pacing [11,12].

In clinical practice, however, many patients do not undergo the recommended echocardiographic optimization process because of the lack of availability of skilled staff time. This may explain why the uptake of optimization is low; in one survey only 45% of patients received AV delay optimization [17]. Uptake is likely to remain low until a more technically convenient method is available. It is difficult for the echocardiographic method to be automated because it requires an operator to keep the probe still while different settings are tested, and professional judgement is needed to decide upon the preferred patterns. Blood pressure has the advantage over Doppler velocity of being able to undergo numerous replicate measurements without skilled operators making the measurements. We have reported on methods for conducting pressure-based optimization which produces an unambiguous optimum, is reproducible, can be automated, and recommends biologically plausible values [14,18,19].

### Design of echocardiographic protocol

In the CARE-HF study AV delay was optimized using Doppler echocardiography of trans-mitral flow using the iterative method [20]. The AV delay which was found to provide maximum separation of the E and A waves was programmed. This method for AV delay optimization is often recommended in guidelines [21]. Estimation of stroke volume using echocardiography, is recommended as the method of choice for performing VV optimization in guidelines for echocardiographic optimization [21].

### Design of haemodynamic protocol

Outside the heart, the most readily observable effects of changes in efficacy of CRT are changes in pressure and flow. Of these pressure is more straightforward to measure non-invasively with automatic equipment.

We have previously demonstrated that changing AV delay has a larger effect on haemodynamics than changing VV delay and results in a curvilinear acute blood pressure response, which fits closely to a parabola [22]. A superior signal to noise ratio is seen when this is performed at higher heart rates [14]. Of the components of blood pressure, systolic blood pressure appears to be the most informative and reproducible when used for AV delay optimization [18]. The algorithm requires multiple alternations between a tested and reference delay and comparisons are made immediately before and after the change [23]. This method is amenable to performing large numbers of replicates, which narrows the confidence interval of the estimated optimum [24]. We have found that it is possible to closely predict the exercise optimum from resting measurements by pacing at different heart rates [25]. Very recently we showed how this could be done with an appropriately transformed signal from a pulse oximeter [26].

In BRAVO (British randomised controlled trial of AV and VV optimization) we adopted a standardised protocol based on our previous reports, which we describe in the methods below. In principle, it could be carried out with invasive pressure or with non-invasive pressure or pulse oximetry recorded beat-to-beat with any appropriate sensor.

The BRAVO study was set up with the aim of determining whether optimization using non-invasive haemodynamics is a possible clinical alternative to the recommended echocardiographic optimization. If the clinical outcomes are equivalent, this method would offer important logistical advantages over echocardiography as it can be operated by a single operator, could be automated, causes minimal discomfort to the patient, and the technology could be developed to become integral to new CRT devices. BRAVO is designed as a non-inferiority study with the primary endpoint of objective exercise capacity measured using peak oxygen uptake (peak VO_2_). Secondary endpoints are symptoms, ventricular volumes, and NT-proBNP.

## Study design

Patients will be recruited from 20 centres in the United Kingdom. The trial has a crossover design. Patients are randomly allocated to have the settings of their device optimized using one method for six months and then the other for six months.

**Figure 1:**
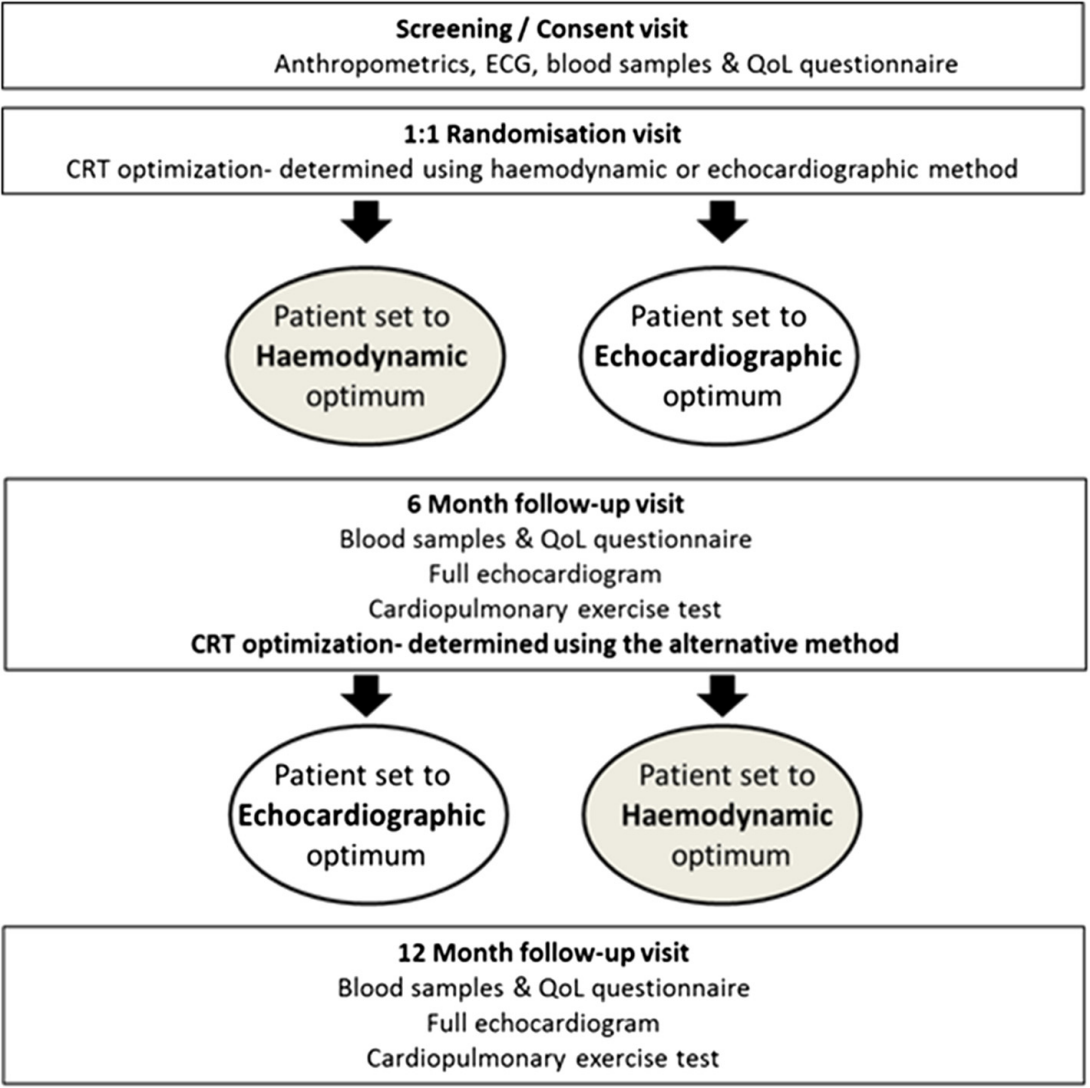
**Flowchart of study design**. Patients visit 4 times and undergo 2 AV/VV delay optimizations according to the echocardiographic and finometer protocols.

A flow chart outlining the study design is provided in Figure 1. At the screening visit, the inclusion and exclusion criteria are checked and informed consent is obtained from those who meet the criteria for entry.

Patients are then randomized. Each patient then undergoes optimization according to their randomized arm. Patients in sinus rhythm have both AV and VV optimization performed. Patients in atrial fibrillation undergo VV optimization only. Rate adaptive AV delay functions are not activated during the study.

After 6 months patients attend for assessment including the primary and secondary endpoints of cardiopulmonary exercise, echocardiographic assessment of LV volumes, QOL questionnaire, and NT-pro BNP. They then crossover to the other form of optimization, and have their pacemaker set to this programming for the next six months. After six months in the second arm, they attend again for assessment of the endpoints. They have now completed the study. Those patients who have a preference for one of the two sets of settings will have the device programmed according to their preference. For the remainder, we will programme the pacemakers according to the preference of their primary cardiologist.

The study design is Prospective, Randomized, and Open with Blinded evaluation of Endpoints (PROBE). The analysis of the cardiopulmonary exercise test data, echocardiography, and blood results are performed by investigators blinded to the study arm.

### Inclusion and exclusion criteria

Study inclusion and exclusion criteria are shown in Table 1. Patients must have a previous diagnosis of symptomatic chronic heart failure and have been implanted with a biventricular pacemaker or biventricular defibrillator device. The device must have been implanted at least six months prior to enrolment, because cardiac reverse remodelling is known to occur after implantation and one of the secondary outcome measures is left ventricular volumes. We wanted to minimize the potential for any differential effect on remodelling of the two optimization schemes to be interfered with by effects arising from recent implantation of the device.

**Table 1:** Inclusion and exclusion criteria

***Inclusion criteria***	***Exclusion criteria***
A previous diagnosis of chronic heart failure	Major cardiovascular event within 6 weeks prior to enrolment
Cardiac Resynchronisation Therapy Device (CRT) implanted at least six months prior to enrolment.	Uncontrolled hypertension
A history of symptomatic congestive heart failure (NYHA Class II to IV).	Participation in any other clinical trial which would conflict with this trial
At any time in the past, an ejection fraction <40% or documented moderate to severely impaired systolic dysfunction.	Any condition that will preclude them from walking on a treadmill adequately or participating fully in the study
Stable medical therapy for heart failure	
The patient is willing, able, and committed to participate in the study for the full length of the follow-up.	
>90% biventricular pacing at entry	

Since the primary endpoint is peak VO_2_ measured using a treadmill cardiopulmonary exercise test, ability and willingness to walk on a treadmill is an inclusion criterion.

### Endpoints

The endpoints of BRAVO are designed to assess physiological markers which are consistently reported to improve following CRT implantation. The rationale for this is that if these markers are less favourable with haemodynamic optimization than with standard echocardiographic optimization, then there would be good reason to reject the use of this haemodynamic optimization since it is biologically implausible that longer term outcomes would be satisfactory when these physiological markers were worse.

### Primary endpoint

The primary endpoint is objective exercise capacity defined as peak VO_2_ on cardiopulmonary exercise testing [27].

Peak VO_2_ measures a physical capacity that integrates many elements and has strong prognostic value in heart failure [28] Reproducibility of repeated measurements of peak VO_2_ at separate hospital visits is well defined [29] and measurement variability is sufficiently low to allow it to be a suitable measure for assessing the impact of optimization of CRT device settings on exercise capacity without requiring an excessively large study population.

### Secondary endpoints

Secondary outcome measures are left ventricular reverse remodelling, quality of life score and N-terminal pro B-type Natriuretic Peptide (NT-pro BNP). Left ventricular end diastolic volume (LVEDV) is measured by echocardiography and has been reported to decrease after implantation of CRT devices [30].

Quality of life is assessed using standardised scores for heart failure: SF-36v2 [31] and Minnesota living with heart failure score [32].

The third secondary endpoint is NT-pro BNP. Neuroendocrine activation may be both a marker of and a mechanism for progressive deterioration in cardiac function [10,33].

### Study conduct and regulatory issues

This study is conducted in compliance with Good Clinical Practice (GCP) guidelines, consistent with the most recent version of the Declaration of Helsinki. The study has been approved by the South West London Research Ethics Committee (3) and site specific assessments were performed for each participating hospital.

### Clinical testing centres

While patients are recruited from many different hospitals, all study visits are performed at one of two testing centres. The two testing centres are Aberdeen Royal Infirmary and Imperial College London. The advantage of this approach is that the equipment for making clinical measurements is kept constant during the study. It also facilitates training of staff performing the study in order to keep study costs to a minimum.

### Measurements

#### Baseline measurements

All recruited patients undergo a baseline physical examination, medical history questionnaire, 12 lead ECG including a documentation of the underlying rhythm where possible, anthropometrics, blood samples are taken and QoL score documented as described below.

### AV and VV delay optimization

Patients in sinus rhythm have the following AV delays tested unless the rhythm becomes intrinsic at a shorter AV delay: 40, 80, 120, 160, 200, 240, 280, and 320 ms. Some devices do not permit 240 and 280 ms: in which case 250 and 275 ms are used instead.

Tested VV delays are LV first by 80 ms, 60 ms, 40 ms, 20 ms, 0 ms, and RV first by 20 ms, 40 ms, 60 ms and 80 ms. In devices that do not allow programming of these values, the nearest permitted value is used.

### Non-invasive haemodynamic optimization protocol

Beat-to-beat blood pressure is measured non-invasively using the Finometer device (Finapres Medical Systems, Netherlands). This digital photoplethysmograph uses an inflatable finger cuff and applies a volume-clamp technique [34] to yield a continuous arterial pressure waveform. This technique has been extensively validated for measuring changes in blood pressure [35-38].

The data is read into a custom data acquisition system. In brief, this consists of an analogue-to-digital card (DAQCard-6062E, National Instruments, Austin, TX, USA) and workstation running custom-written software in the Labview instrument control language (v7.0, National Instruments, Austin, TX, USA). Data is analysed off-line using custom-written software [39-41] based largely on Matlab (Natick, MA, USA), which has been developed, and validated at the lead centre [41-43].

When using a physiological variable in order to guide the optimization of CRT settings it is important that enough steps are taken to minimize the effects of biologic variability. Without these steps noise is likely to overwhelm the signal and the results obtained are unlikely to be reliable [44,45].

**Figure 2:**
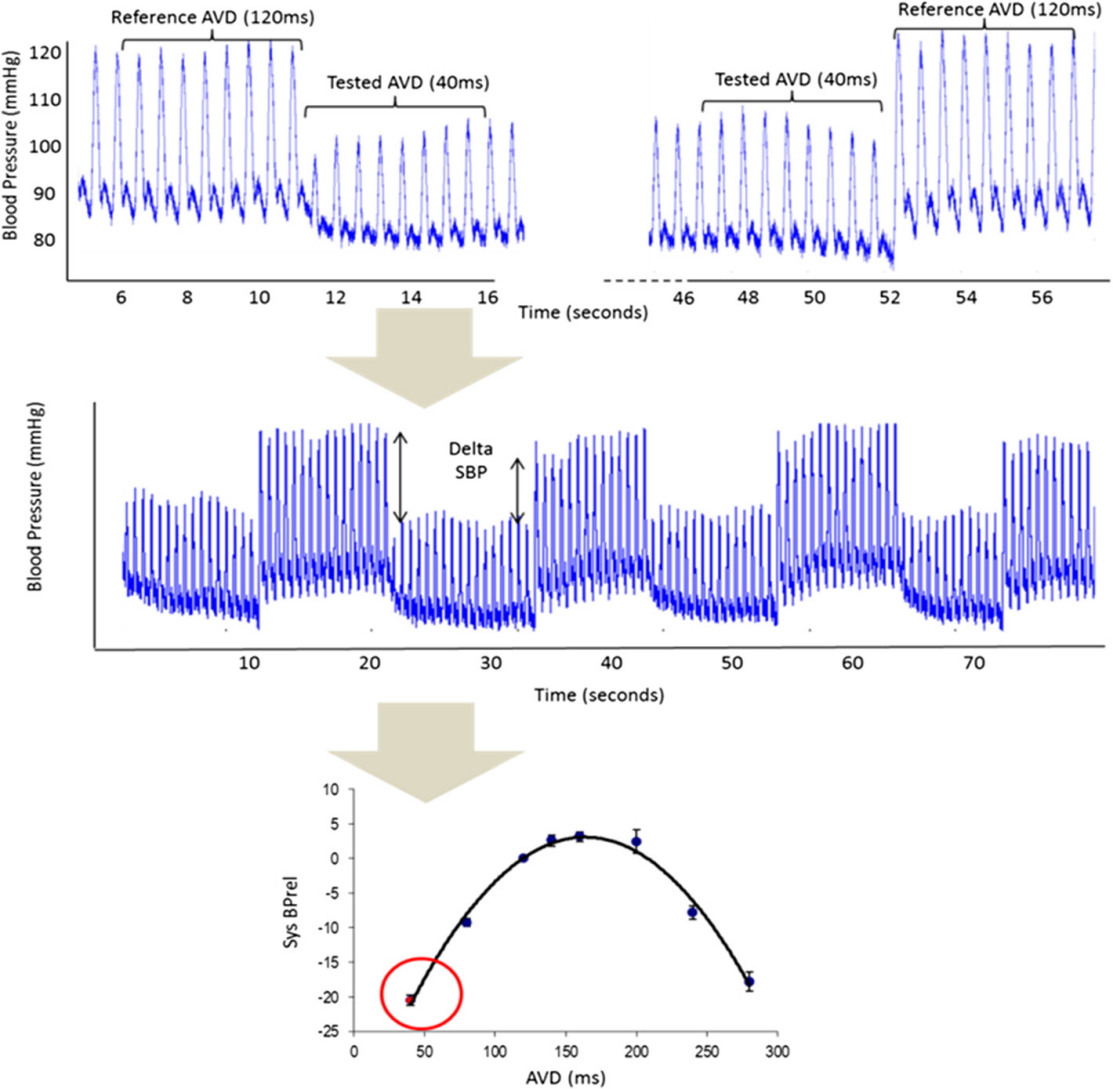
**Calculating the relative change in systolic blood pressure (Sys BPrel (mmHg) between a reference atrio-ventricular delay (AVD) of 120 ms and a tested AVD of 40 ms.** Eight beats from the continuous arterial pressure waveform are averaged from each AVD (top picture), this process is repeated to include a minimum of 6 transitions for each AVD tested (middle picture). SBPrel plotted against the AVD (lower picture) is an average of the change in systolic blood pressure (8-beat average).

In this study we use a protocol designed to minimise the effect of noise, this has been described in detail previously but in brief consists of the following steps [14,22,23,25]. Each tested delay is compared to a reference delay (for AV delay 120 ms, for VV delay 0 ms) and the relative change in acute blood systolic blood pressure is calculated as the difference of the mean of 8 beats immediately before the transition to the tested delay compared to the mean of the 8 beats immediately after the transition. For each tested delay multiple transitions between the tested and reference delays (a minimum of six transitions) are made and the mean relative change in acute systolic blood pressure is calculated (Figure 2).

The optimal AV or VV delay is identified as the peak of a fitted curve [22] unless the longest tested delay results in the greatest increase in pressure, in which case this is taken as the optimal value since fitting a curve in this case will result in a non-physiological AV delay being reported as optimal.

In order to maximize signal-to-noise ratio, optimization is performed during atrial pacing with an elevated heart rate (40 bpm above the resting heart rate if paced or 45 bpm if sensed). This identified optimum is programmed as the paced AV delay. The sensed AV delay for haemodynamic optima is programmed to be 60 ms shorter based on data previously acquired with this protocol.

### Echocardiographic optimization protocol

**Figure 3:**
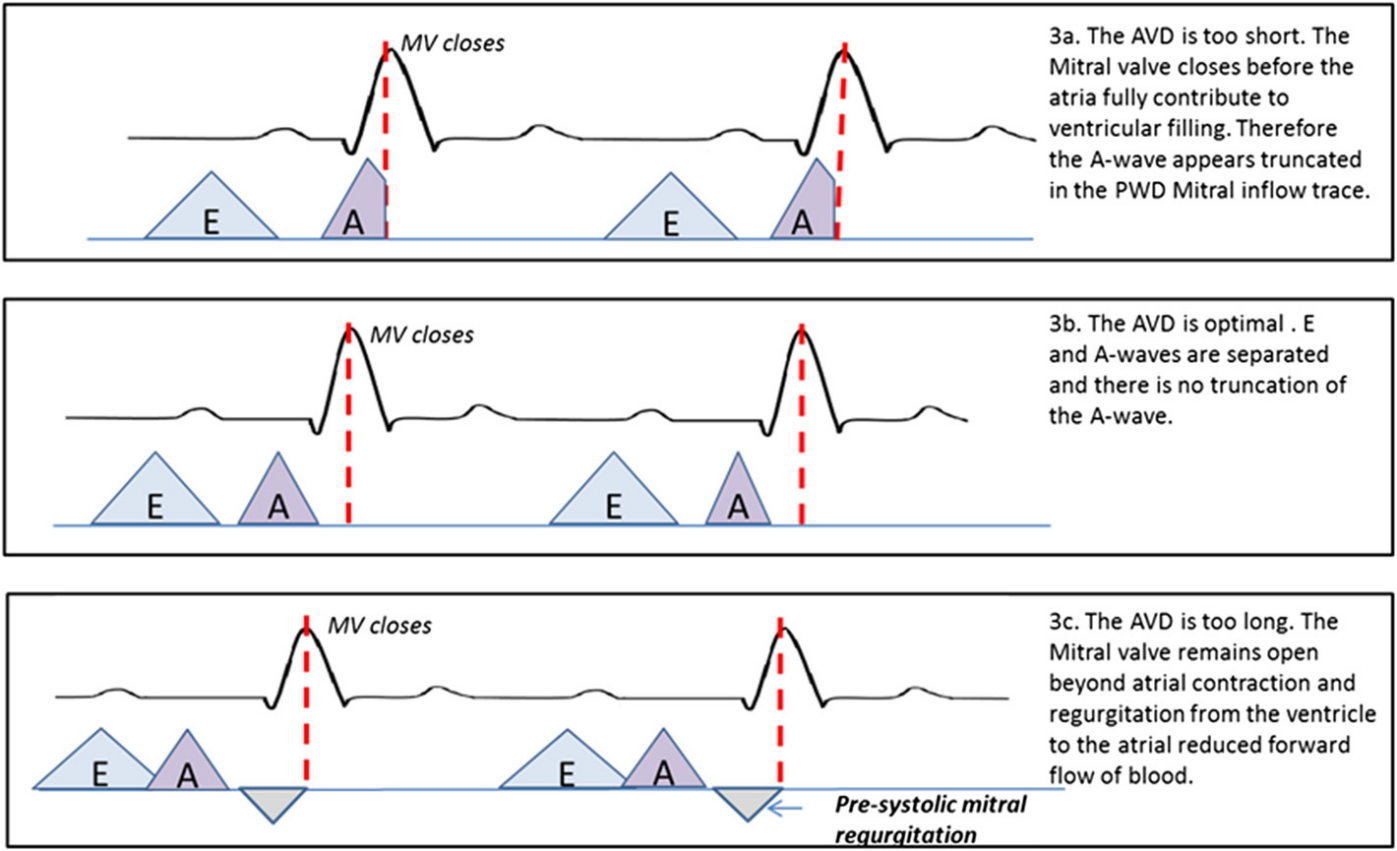
**Echocardiographic optimization of atrio-ventricular delay (AVD) using the iterative method.** Abbreviations E wave, A wave.

AV optimization is performed by assessing the pattern of pulsed wave Doppler recorded through the mitral valve using the iterative method (Figure 3) [11]. Doppler data is acquired using a GE Vivid-I machine (GE Healthcare). Delays which produce intrinsic ventricular conduction are excluded from analysis. The AV delay with the best filling pattern, defined using the published protocol from CARE-HF, [11] is programmed as the sensed AV delay. The paced AV delay is programmed as 30 ms longer than the optimal sensed AV delay as per recent guidelines [21].

VV optimization is performed after AV delay optimization. The AV delay is set to the value determined as optimal during the AV delay optimization during VV optimization. The VV delay yielding the highest mean LVOT VTI, determined from 5 beats, is selected as the optimal VV delay.

### Endpoint measurements

#### Cardiopulmonary exercise testing

Patients exercise on a treadmill using a “smoothed modified Bruce” protocol [46]. Spirometry and gas exchange are monitored using the COSMED Quark CPX System (COSMED S.r.l. Rome, Italy) or an Medisoft Ergocard ++ unit (Medisoft group, Sorrines, Belgium), Exercise capacity is measured as peak VO_2_ in ml/kg/min. VE/VCO_2_ slope and oxygen uptake efficiency slope are also measured [29].

### Echocardiography

Additional echocardiographic measurements are made using a Philips ie33 machine (Philips, Netherlands) using an S5-1 probe with the patient semi recumbent in the left lateral position. For each parameter the mean of three measurements are calculated, the following parameters are measured: left ventricular dimensions, volumes and ejection fraction from the four and 2 chamber views, quantification of mitral regurgitation and maximum velocity through the aortic valve. Tissue Doppler imaging (TDI) is used to measure longitudinal function at the mitral valve annulus level on the lateral, septal, and inferior walls. Where image quality is suitable a 3D dataset is obtained using an X3-1 probe and used for off-line calculation of volumes and ejection fraction by a physiologist blinded to the study arm.

### Quality of life questionnaire

Quality of life (QoL) is scored using the Minnesota Living with Heart Failure Questionnaire (MLHFQ) (http://www.mlhfq.org/) [32] and SF-36 [31] (http://www.sf-36.org/tools/sf36.shtml). The MLHFQ questionnaire is a validated condition-specific measure with good sensitivity and discrimination. It will be complemented with the SF-36, a validated generic measure of QoL, which enables comparison of findings with other outcome studies.

### Blood samples

Blood samples are taken for analysis of N-terminal pro-B-type Natriuretic Peptide (NT-pro BNP).

### Sub-studies

In a subset of patients reproducibility of cardiopulmonary exercise testing is investigated by conducting a second exercise test, within a two week period, prior to programming the AV delay. Sub-studies are also performed to assess the reproducibility of the two optimization protocols. In a further subgroup the impact of AV delay on longer term blood pressure is assessed by 24 hour ambulatory blood pressure measurement. This is carried out, following optimization, at the initial visit (time of randomization) and again at 6 month follow- up.

### Statistics

Distributions will be described by the mean and standard deviation, where they meet criteria for normality. Skewed data will be appropriately transformed prior to analysis. Analysis of the cross-over data will be performed using linear mixed effects models [47]. Sample size calculations were carried out using nQuery Advisor and are given in detail in the section below.

### Sample size calculations

All sample size calculations were based on paired t-tests comparing outcomes for echocardiographic optimization versus haemodynamic optimization.

The reproducibility of peak VO_2_ in heart failure patients between two separate visits using a smoothed modified Bruce protocol on a standard treadmill is 2.4 ml/kg/min, expressed as standard deviation of difference between two independent measurements [48]. Implantation of a CRT pacemaker results in a 0.5-2.5 (mean 1.5) ml/kg/min increase in peak VO_2_[8,9,49-53]. Simply implanting a CRT device yields a haemodynamic improvement of 5–6 mmHg of systolic blood pressure [11,12]. Optimizing the settings after implantation appears in preliminary work to add a further 3-6 mmHg increase in pressure, [1] i.e. between 50-100% of the effect of CRT.

The study sample size was chosen to have 90% power to detect a margin of equivalence of 0.75 ml/kg/min at the 5% significance level. This is the smallest difference between the treatment mean and the reference mean that still results in the conclusion of non-inferiority and corresponds to half the expected clinically meaningful difference as advocated by Jones *et al*. [54] using a one sided *t*-test of equivalence in means. On this basis 338 patients (N1 = 177/N2 = 177) are required. Mean mortality and dropout in similar populations over a 12-month period has been observed to be between 10% and 15%. In order to accommodate possible losses to follow-up as high as this, the target enrolment is 400 patients.

### Left ventricular end-diastolic volume

The standard deviation of the difference of LV end-diastolic volume measurements has previously been reported as 23.8 ml [55]. Following CRT reductions in LV end-diastolic volumes are variable but of the order of 25 ml [56] On this basis 338 patients provides >99% power to detect a margin of equivalence of 12.5 ml (50% of clinically significant difference), and permits a difference of 7.4 ml to be detected with 90% power at a significance level of 5%.

### Quality of life and NT-pro BNP

For SF-36, the standard deviation of the difference of scores has previously been reported to be 26 [17]. Previous studies of CRT have reported improvements of ~17. With 338 patients we have 92% power to detect a margin of equivalence of 8.5 at a significance level of 5%. For the Minnesota questionnaire, mild improvements in QoL are represented by a change of 12 with a typical standard deviation of 15 [48]. With 338 patients we would be able to easily detect a mild improvement of this magnitude (>99% power) and have 90% power to detect an equivalence limit of 4 points in the Minnesota score.

For NT-proBNP, CARE-HF reported median reductions of 656 pg/ml in NT-pro BNP (adjusted for change in control group) [10]. The within subject coefficient of variation (σ_w_/mean) in log NT-proBNP levels in people with chronic heart failure over 12 months has been reported to be ~10%, i.e. around 190 pg/ml [57]. Assuming a log normal distribution of NT-proBNP, with 338 patients we will be able to easily detect an equivalence limit of half the clinically reported difference with >99% power at 5% significance level and have 90% power to detect an equivalence limit of 0.062 log units (i.e. a fall in baseline NT-pro BNP from 1900 pg/ml to 1647 pg/ml) at a 5% significance level.

### Adverse events

All adverse events will be documented. Serious adverse events are defined as those which are not anticipated or not known to be related to the condition being studied or the intervention being used that would result in any of the following outcomes: death, life-threatening condition, unexpected/unplanned in-patient hospitalisation, persistent or significant disability or incapacity, is a congenital anomaly or birth defect.

### Timelines

The first patient was randomised in December 2010. Recruitment is expected to be complete by December 2013. The study will report in 2015 after follow up of all participants is complete.

## Discussion

If exercise capacity is non-inferior with haemodynamic optimization compared with echocardiographic optimization, it would be proof of concept that haemodynamic optimization is an acceptable alternative which has the potential to be more easily implemented. We already know that it is much more capable of being automated, and therefore consumes less expert time. This would then allow more patients to have their resynchronisation devices optimised and allow them to reap maximum benefit from their devices.

A favourable result in this study could stimulate worldwide change in practice by providing a new means for improving patient’s symptomatic and physiological outcomes using existing devices.

## Competing interests

The authors declare that they have no competing interests.

## Authors’ contributions

ZIW was involved in conception and design of the study, and analysis of the data. SMAS is involved in recruitment, data collection, and analysis. SJ is involved in recruitment, data collection, and analysis. KM is involved in recruitment, data collection, and analysis. EC is involved in coordinating the study recruitment, and data analysis. AK was involved in design of the study, recruitment, and data collection. JM was involved in the conception and design of the study. ADH was involved in the conception and design of the study. MF was involved in the conception and design of the study. DPF was involved in the conception and design of the study, and analysis of the data. All authors contributed to the drafting of the manuscript, and read and approved the final manuscript.

## Pre-publication history

The pre-publication history for this paper can be accessed here:

http://www.biomedcentral.com/1471-2261/14/42/prepub
